# P-788. Urinary Tract Infection Treatment Failure Differences in a 1:1 Matched Cohort of US Female Patients with and without Diabetes

**DOI:** 10.1093/ofid/ofaf695.999

**Published:** 2026-01-11

**Authors:** Pamela Kushner, Seth Kuranz, Virginia Noxon-Wood, Meghan E Luck, Darrian Tattoli, Tien-Huei Hsu, Jeffrey J Ellis

**Affiliations:** University of California Irvine Medical Center, Orange, CA, United States, Orange, California; Inovalon, Bowie, MD, United States, Bowie, Maryland; Inovalon, Bowie, MD, United States, Bowie, Maryland; GSK, Brattleboro, VT; GSK, Collegeville, PA, United States, Collegeville, Pennsylvania; GSK, Collegeville, PA, United States, Collegeville, Pennsylvania; GSK, Brattleboro, VT

## Abstract

**Background:**

Treating urinary tract infections (UTIs) in patients with diabetes mellitus (DM) is more challenging than in those without DM. We compared treatment failure (TF) occurrence in female patients with/without DM who were treated for otherwise uncomplicated UTI (uUTI).

**Figure:**
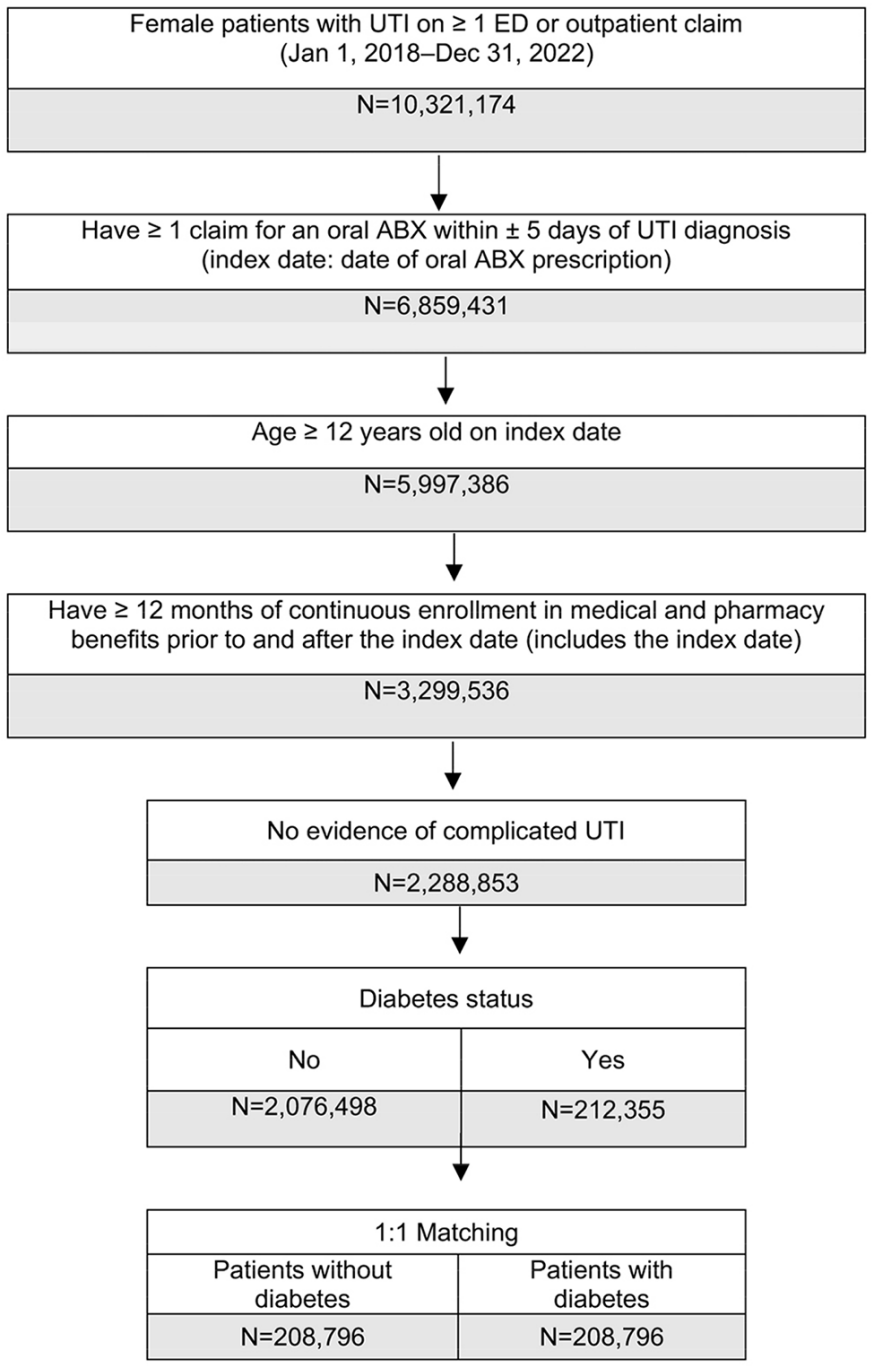

**Figure:**
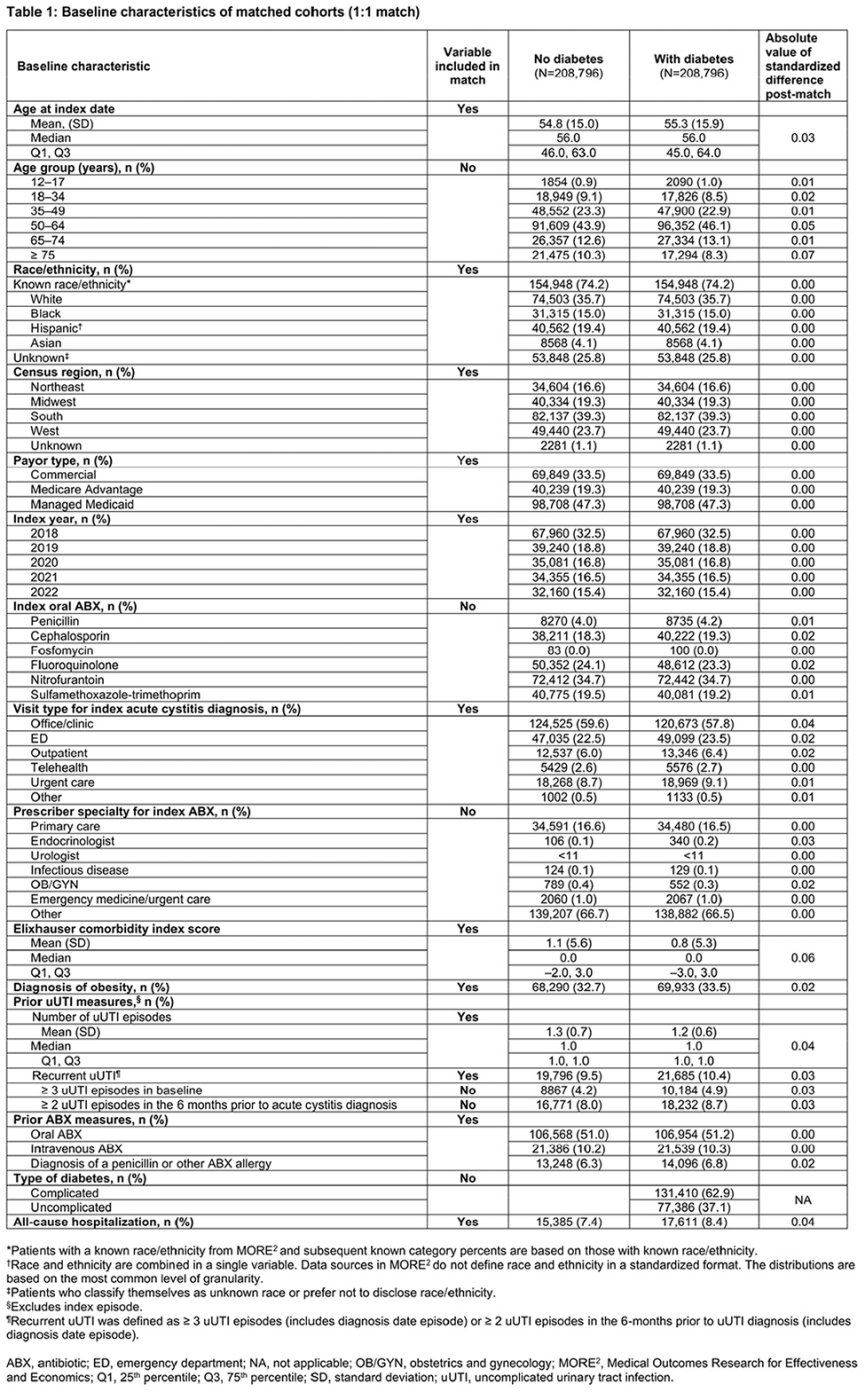

**Methods:**

This observational, retrospective analysis of the real-word deidentified Inovalon MORE^2^ Registry included female patients (≥ 12 years) with a uUTI diagnosis (Dx) Jan 2018–Dec 2022. Index date was the first oral antibiotic (ABX) claim observed ± 5 days of uUTI Dx. Eligible patients with Type 1/Type 2 or other DM had either of the following observed in the 12 months prior to the index date: ≥ 1 claim with a Dx of DM and ≥ 1 claim for an antidiabetic treatment, or ≥ 2 claims with a Dx of DM. TF was defined as having a second oral ABX, intravenous ABX, or emergency department or inpatient stay with a primary Dx of UTI ≤ 28 days post index date. A 1:1 greedy propensity score match (PSM) without replacement and a caliper of 0.1, with direct matching on index year, race/ethnicity, region, and payer type, was utilized to match DM and non-DM cohorts. Standard differences (std diff) were used to assess DM versus non-DM cohort baseline characteristics; values < 10% indicated a good match and balance. Univariate comparisons between matched DM and non-DM cohorts were conducted using Z-tests for proportions and t-tests for means.

**Figure:**
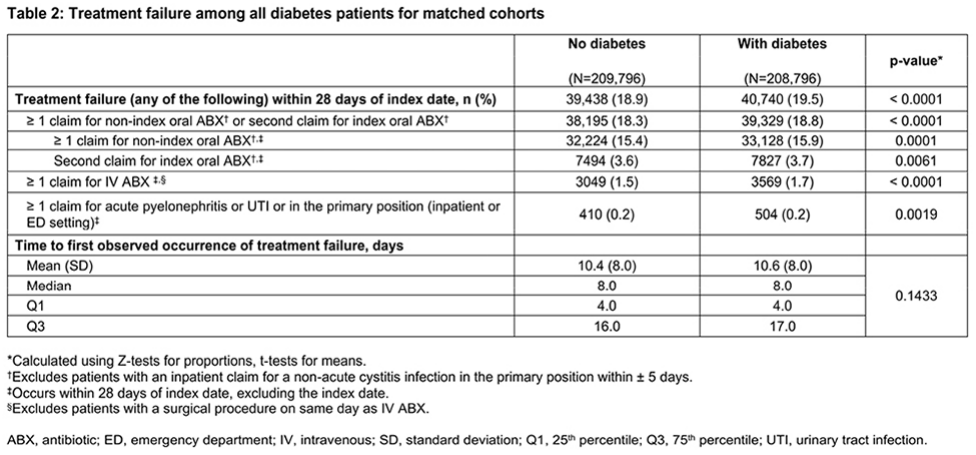

**Results:**

Overall, 2,076,498 and 212,355 patients were identified without DM and with DM, respectively. The PSM retained 208,796 patients in the DM cohort matched 1:1 with patients in the non-DM cohort (Figure). Patient characteristics were well matched (all std diff < 0.10; Table 1). Mean (median) ages for non-DM and DM cohorts were 54.8 (56.0) and 55.3 (56.0) years, respectively. TF occurred in 18.9% and 19.5% of the non-DM and DM cohorts, respectively (p < 0.0001; Table 2). Mean (median) time to first TF was 10.4 (8.0) days in non-DM and 10.6 (8.0) days in DM cohorts (p=0.1433). The need for a second oral ABX was the predominant indicator of TF for both cohorts.

**Conclusion:**

While TF was statistically higher in patients with DM versus those without DM, the small numerical difference challenges its clinical relevance. Future studies should assess impact of DM control on TF compared to patients without DM.

Funding: GSK study 222865.

**Disclosures:**

Pamela Kushner, MD, GSK: Advisor/Consultant|GSK: Speaker Seth Kuranz, PhD, Inovalon: Employee of Inovalon, a consulting company that received funding from GSK to conduct this study. Virginia Noxon-Wood, PhD, Inovalon: Employee of Inovalon, a consulting company that received funding from GSK to conduct this study. Meghan E. Luck, PharmD, GSK: Employee|GSK: Stocks/Bonds (Public Company) Darrian Tattoli, PharmD, GSK: Employee|GSK: Stocks/Bonds (Public Company) Tien-Huei Hsu, PhD, GSK: Employee|GSK: Stocks/Bonds (Public Company) Jeffrey J. Ellis, PharmD, MS, GSK: Employee|GSK: Stocks/Bonds (Public Company)

